# Thoracobiliary Fistulae: Diagnostic Challenges and Surgical Solutions in Two Cases

**DOI:** 10.7759/cureus.96839

**Published:** 2025-11-14

**Authors:** Abdul Kamil Ghumman, Muhammad Ayzed, Muhammad Khawar Shahzad, Tariq Ali Bangash, Muhammad Hamza Laique

**Affiliations:** 1 Hepato-Pancreato-Biliary and Liver Transplant Surgery, Shaikh Zayed Hospital Lahore, Lahore, PAK

**Keywords:** biliary tract diseases, complications of hepatobiliary surgeries, hepatobiliary surgical procedures, hydatid cyst complications, thoracobiliary fistula

## Abstract

Thoracobiliary fistulae (TBF) are rare and highly morbid conditions characterized by communication between the biliary tract and the pleural cavity or bronchial tree. The vague nature of symptoms results in delays in diagnosis, and there are no standardized treatment guidelines. Here, we share the experiences of two patients treated at the Hepatobiliary Unit, Shaikh Zayed Hospital, Lahore. The first case developed a bronchobiliary fistula after a right hemihepatectomy for a hydatid cyst, while the second developed a biliopleural fistula following hepaticojejunostomy stenosis, performed for a common bile duct injury. Both patients were evaluated with advanced imaging (CT, magnetic resonance cholangiopancreatography (MRCP), and endoscopic retrograde cholangiopancreatography (ERCP)) and ultimately received surgery through an abdominal approach. In both cases, removing the fistulous tract and repairing the liver and diaphragmatic fistulous openings provided full relief of symptoms. The first patient recovered smoothly, while the second experienced transient hepatic decompensation (due to longstanding cholestasis), which improved with supportive care. Early detection of TBF, using bilirubin levels in sputum or pleural fluid and targeted imaging to delineate anatomy, is critical to reducing the risk of complications. For patients who can undergo surgery, excision of the fistulous tract through the transabdominal route is a safe and effective option. Our experience adds to the limited global knowledge on TBF and highlights the need for clear treatment guidelines for this rare but morbid condition.

## Introduction

Thoracobiliary fistulae (TBF) are rare pathological communications between the biliary tree and the pleural cavity (biliopleural fistula) or bronchial tree (bronchobiliary fistula). These fistulae, which were initially reported by Peacock in 1850, are among the most uncommon side effects of hepatobiliary disease [1]. Compared to congenital forms, acquired forms are much more common and are typically linked to liver abscesses, trauma, cancer, hydatid cyst rupture, or prior hepatobiliary surgery [2,3].

Even though biliptysis, or the expectoration of bile-stained sputum, is the clinical hallmark of TBF, other symptoms like fever, cough, and discomfort in the right upper quadrant are frequently nonspecific, which delays diagnosis. There is no agreement on the best diagnostic or treatment strategy due to the rarity of this entity, and the majority of the evidence that is currently available is restricted to individual case reports and series [4,5].

Numerous diagnostic techniques, such as magnetic resonance cholangiopancreatography (MRCP), endoscopic retrograde cholangiopancreatography (ERCP), CT, and, less frequently, bronchoscopy, have been described. Options for treatment include conservative or minimally invasive procedures (like endoscopic or percutaneous biliary drainage) as well as surgical repair. However, management is still very customized because there are no standardized procedures in place [6,7].

Two thoracobiliary fistula cases that were successfully treated at a tertiary hepatobiliary unit in Shaikh Zayed Hospital in Lahore, Pakistan, are presented in this paper. After undergoing intricate biliary surgery, both patients experienced postoperative TBF. As we add to the limited body of knowledge on this uncommon but morbid condition, we hope that this case series will highlight the value of early diagnosis, multidisciplinary planning, and customized surgical management.

## Case presentation

Case 1

A 50-year-old male, with no significant past medical history, underwent a right hemihepatectomy in March 2022 at a private facility for a large hydatid cyst involving the right hepatic lobe. The surgery was complicated by an iatrogenic diaphragmatic injury, which was repaired intraoperatively with the placement of a chest tube. The patient had an uneventful recovery and was discharged on postoperative day 13. Approximately six weeks later, he developed a persistent cough with sputum production and intermittent fever. Over the next two years, he underwent multiple interventional procedures (records unavailable) but continued to suffer from progressive symptoms, most notably chronic biliptysis and copious amounts of bile-stained sputum requiring frequent expectoration, leading to social embarrassment, loss of appetite, and unintentional weight loss of 9 kg (from 65 kg to 56 kg). He presented to Shaikh Zayed Hospital on August 7, 2024, for further evaluation and management. 

On examination, he appeared thin and emaciated (BMI 19 kg/m²), with reduced air entry and basal crepitations over the right lower lung zone. Abdominal examination revealed a scaphoid, non-tender abdomen with a reverse L-shaped surgical scar and no organomegaly. Laboratory investigations, as depicted in Table 1, showed mild anemia (Hb 9.8 g/dl), normal white cell and platelet counts, and elevated alkaline phosphatase (ALP) (252 U/L) with mildly raised bilirubin levels (total 2.1 mg/dl, direct 1.1 mg/dl). Sputum bilirubin was elevated at 1.1 mg/dl. Viral serologies for hepatitis B virus (HBV), hepatitis C virus (HCV), and HIV were negative.

**Table 1:** Laboratory results HB: hemoglobin; TLC: total leukocyte count; PLT: platelet; INR: international normalized ratio; ALP: alkaline phosphatase; AST: aspartate aminotransferase; ALT: alanine aminotransferase; HCV: hepatitis C virus

Parameters	Results	Reference Values
HB	9.8	12-16.5 g/dl
TLC	7.9	4-11 10^9/L
PLT	220	150-450 10^9/L
INR	1.1	0.8-1.1
Bilirubin (Total)	2.1 mg/dl	0.0-1.0 mg/dl
Bilirubin (Direct)	1.1 mg/dl	0.0-0.3 mg/dl
ALP	252 U/L	50-136 U/L
ALT	51 U/L	<63 U/L
AST	75 U/L	<37 U/L
Albumin	3.0 g/dl	3.5-5 g/dl
Anti HCV	Negative	
HbsAg	Negative	
Anti HIV	Negative	
Sputum bilirubin level	1.1 mg/dl	

**Figure 1:**
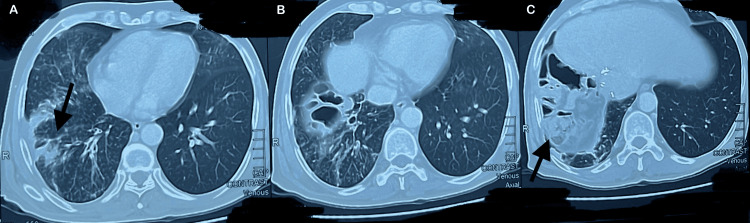
Axial view of CT chest and abdomen with IV contrast A: axial computed tomography (CT) image demonstrating patchy consolidation (arrow) in the right upper/middle lung lobe; B: axial CT image showing marked consolidation and cystic lucencies in the right middle/lower lobe; C: axial CT image at the lung bases and the upper abdomen showing basilar consolidation and pleural thickening in the right lower lobe (RLL). The image confirms the post-operative status of right hemihepatectomy and shows superior migration of bowel loops (arrow) into the liver bed defect.

**Figure 2:**
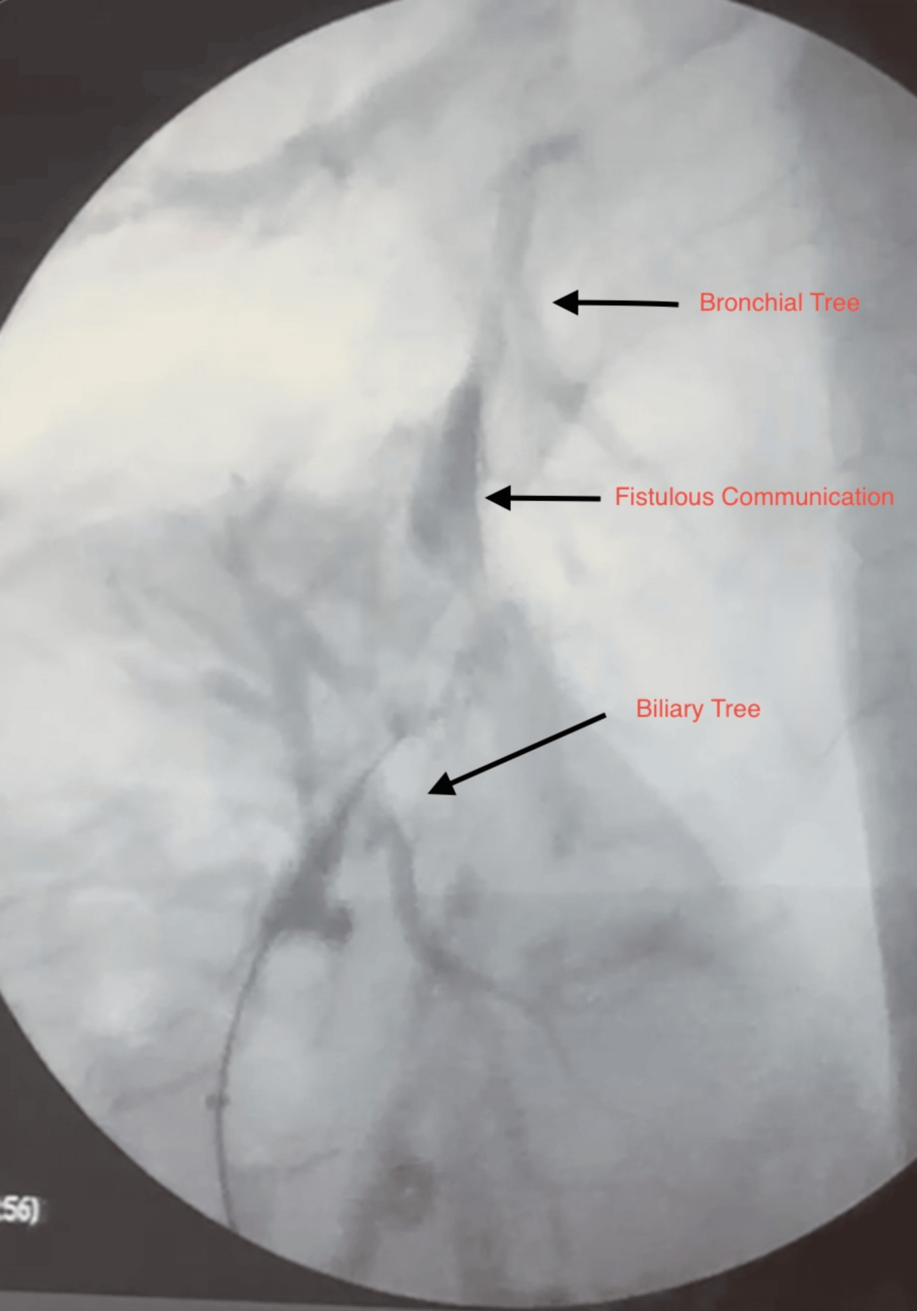
ERCP showing the bronchial tree, biliary tree, and their fistulous communication (arrows) ERCP: endoscopic retrograde cholangiopancreatography

**Figure 3:**
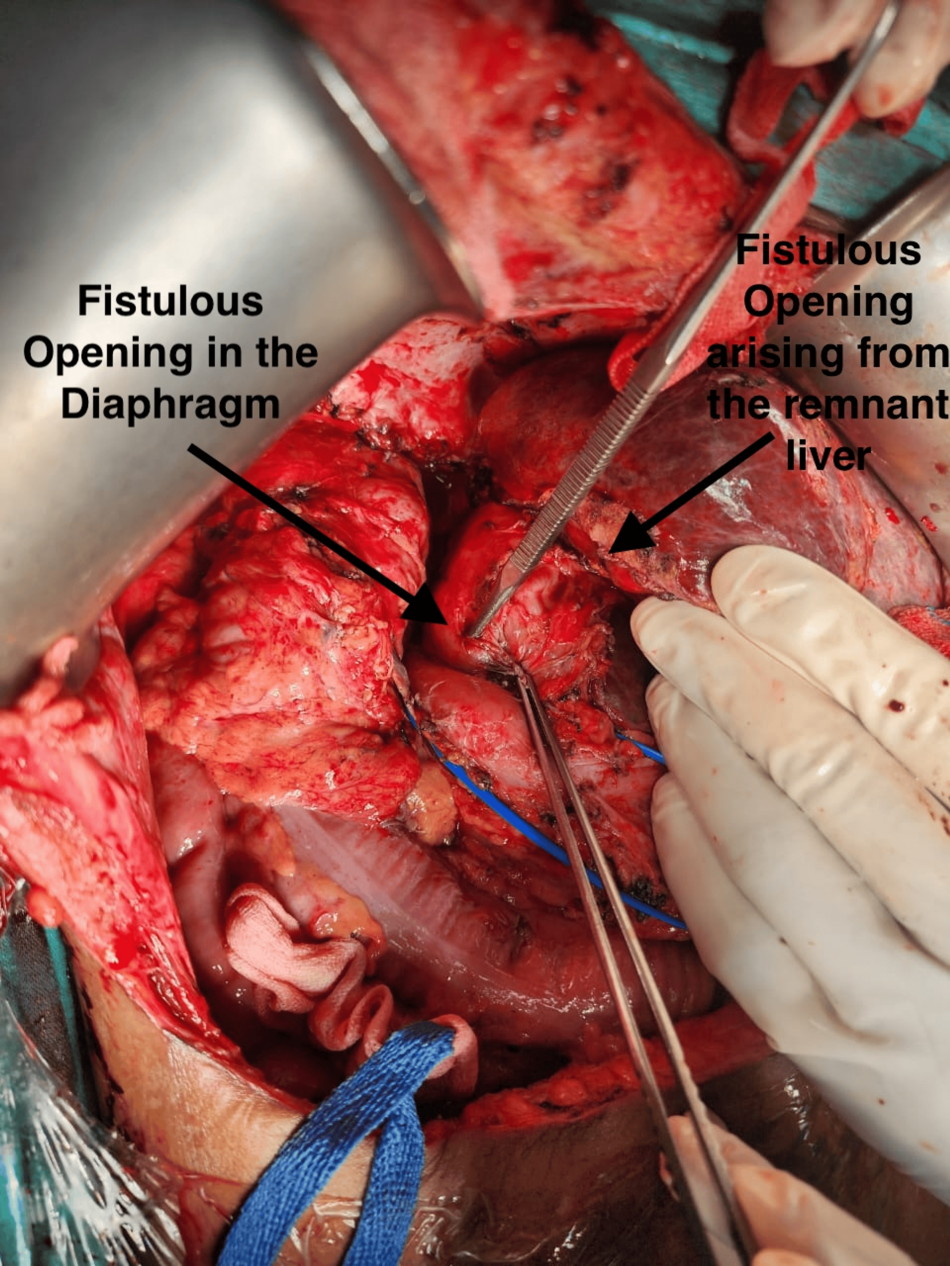
Intraoperative finding showing the fistulous tract originating from the posterior surface of the right hepatic remnant

A CT scan of the chest, abdomen, and pelvis with IV contrast revealed consolidation of the right middle and lower lung lobes and absence of the right hepatic lobe, as shown in Figure 1. MRCP demonstrated a prominent left biliary system with a stricture at the confluence, while ERCP confirmed the presence of a thoracobiliary fistula, as illustrated in Figure 2. The patient underwent an exploratory laparotomy with excision of the fistulous tract and repair of both hepatic and diaphragmatic defects. As shown in Figure 3, intraoperative findings included a fistulous communication originating from the posterior surface of segment 4, traversing the diaphragm into the right bronchial system, confirmed by the presence of bile and air bubbles with colored dye leakage.

Case 2

A 36-year-old Afghan female, with a history provided by her husband due to a language barrier (Farsi-speaking), presented to Shaikh Zayed Hospital on February 10, 2025, with complaints of shortness of breath, dry cough, intermittent low-grade fever, and right-sided pleuritic chest pain ongoing for the past two months. She had a past surgical history of cholecystectomy five years ago at a rural hospital in Afghanistan, complicated by a common bile duct injury, for which she underwent a Roux-en-Y hepaticojejunostomy on January 18, 2020, in Kabul. Her postoperative course had been uneventful until the recent onset of symptoms. Prior to presentation, she had developed a massive right-sided pleural effusion necessitating tube thoracostomy, which had since been continuously draining over 1000 ml/day of greenish fluid (bile). She also reported significant weight loss (35 kg), pruritus, and skin rash. Her medical history was notable for previous episodes of typhoid, malaria, and hepatitis A, and she had received multiple courses of antibiotics over the preceding two months. 

On examination, she was alert, oriented, jaundiced, and in mild respiratory distress with decreased breath sounds on the right hemithorax. A functioning chest tube was in place, with ongoing bile-stained output. Abdominal examination revealed splenomegaly and previous surgical scars, with no tenderness. Laboratory investigations, as shown in Table 2, revealed anemia (Hb 10.2 g/dL), elevated ALP (929 U/L), mildly raised alanine aminotransferase (ALT) (79 U/L), total bilirubin of 3.34 mg/dL, and normal renal function. Pleural fluid bilirubin was markedly elevated at 12.9 mg/dL. 

**Table 2:** Laboratory results HB: hemoglobin; TLC: total leukocyte count; PLT: platelet; INR: international normalized ratio; ALP: alkaline phosphatase; AST: aspartate aminotransferase; ALT: alanine aminotransferase; HCV: hepatitis C virus

Parameters	Results	Reference Values
HB	10.2	12-16.5 g/dl
TLC	8.2	4-11 10^9/L
PLT	394	150-450 10^9/L
INR	1.1	0.8-1.1
Bilirubin (Total)	3.34 mg/dl	0.0-1.0 mg/dl
Bilirubin (Direct)	2.1 mg/dl	0.0-0.3 mg/dl
ALP	929 U/L	50-136 U/L
ALT	79 U/L	<63 U/L
AST	37 U/L	<37 U/L
Albumin	2.8 g/dl	3.5-5 g/dl
S. Creatinine	0.6	0.6-1.3 mg/dl
Anti HCV	Negative	
HbsAg	Negative	
Anti HIV	Negative	
Pleural fluid bilirubin level	12.9 mg/dl	

**Figure 4:**
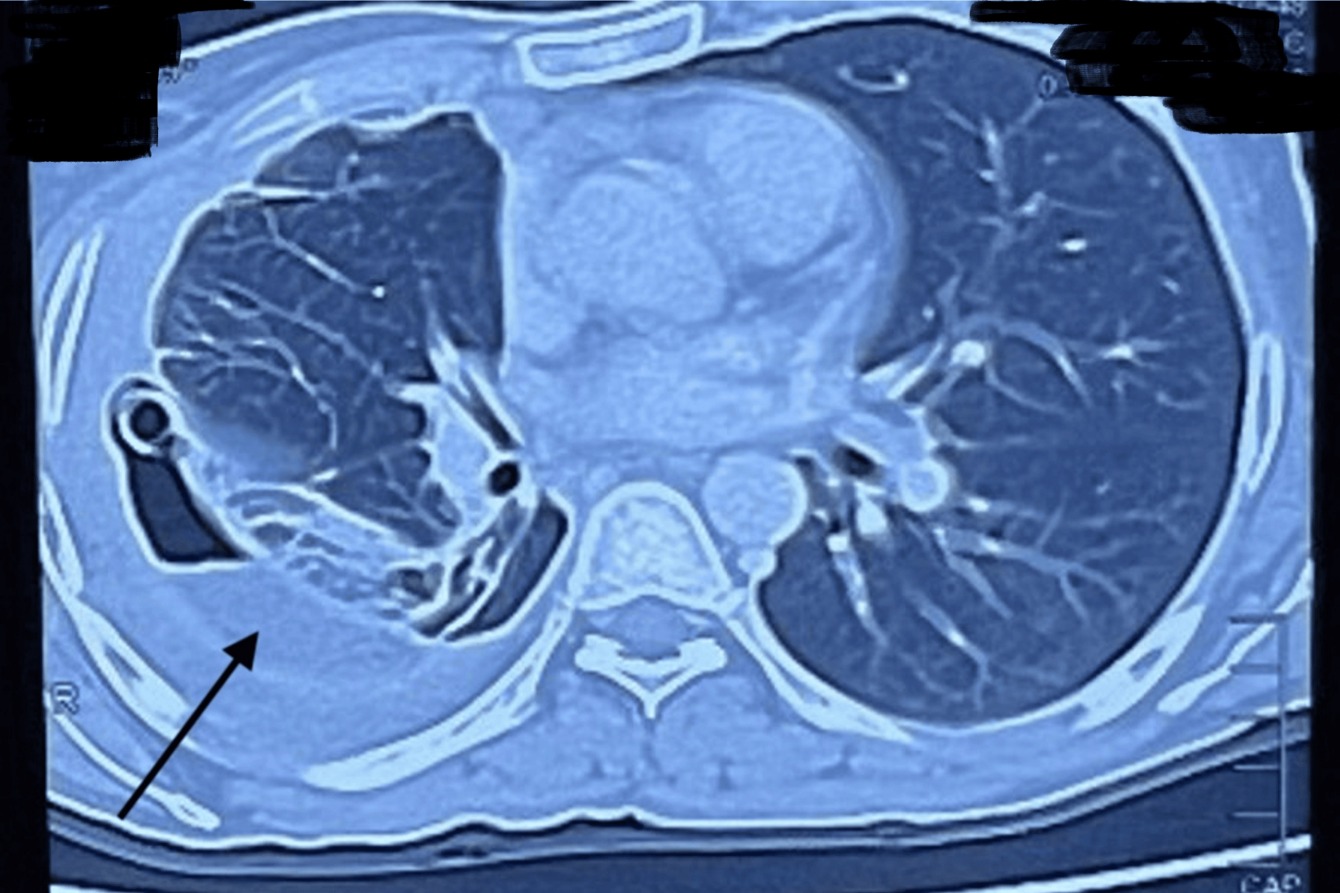
CT chest showing right-sided hydro-pneumothorax with chest tube in situ, compressed lung parenchyma, and basal consolidation (arrow)

**Figure 5:**
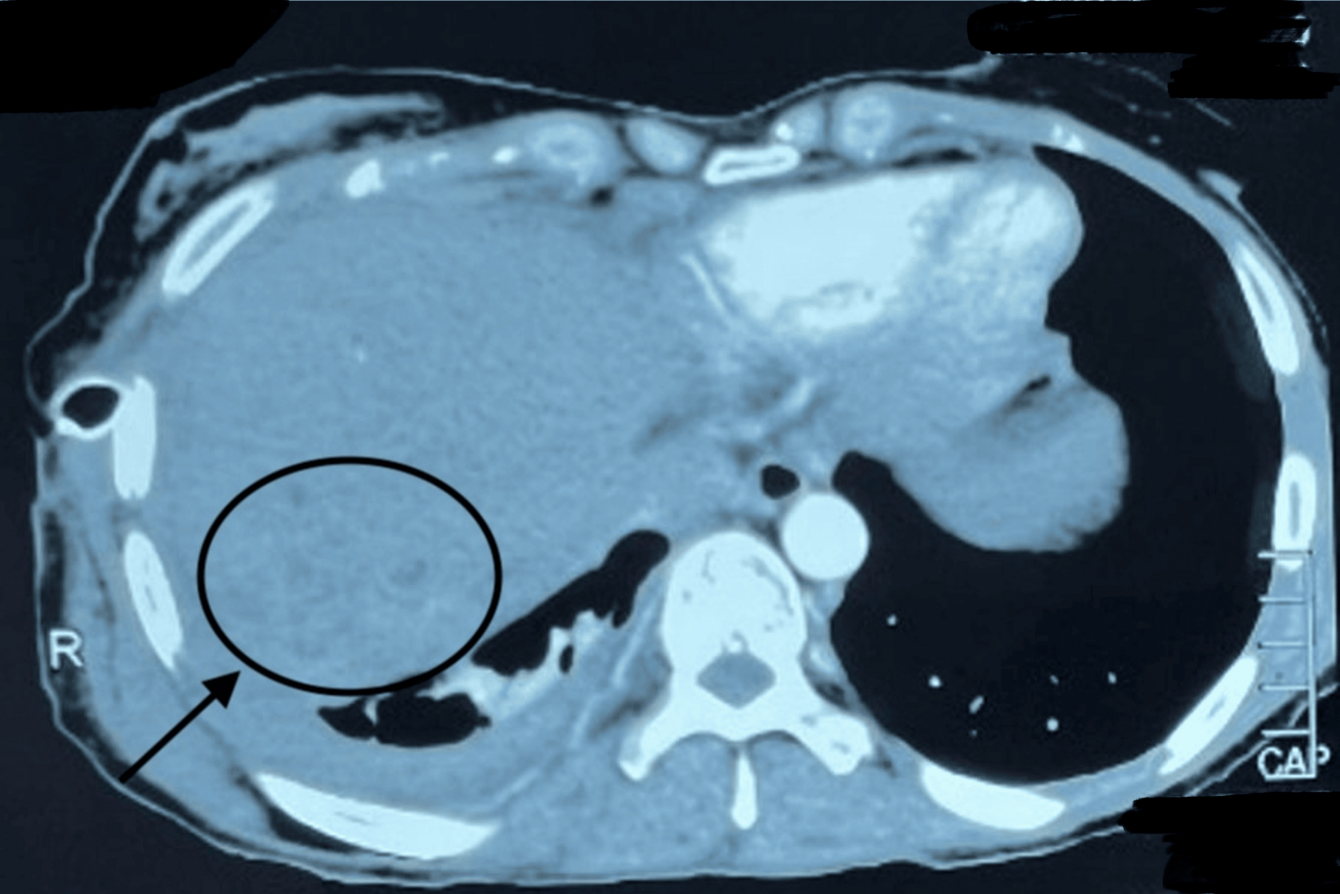
CT abdomen and pelvis with IV contrast showing a segment VII/VIII heterogeneous density area (arrow) possibly communicating with the adjacent pleural cavity.

**Figure 6:**
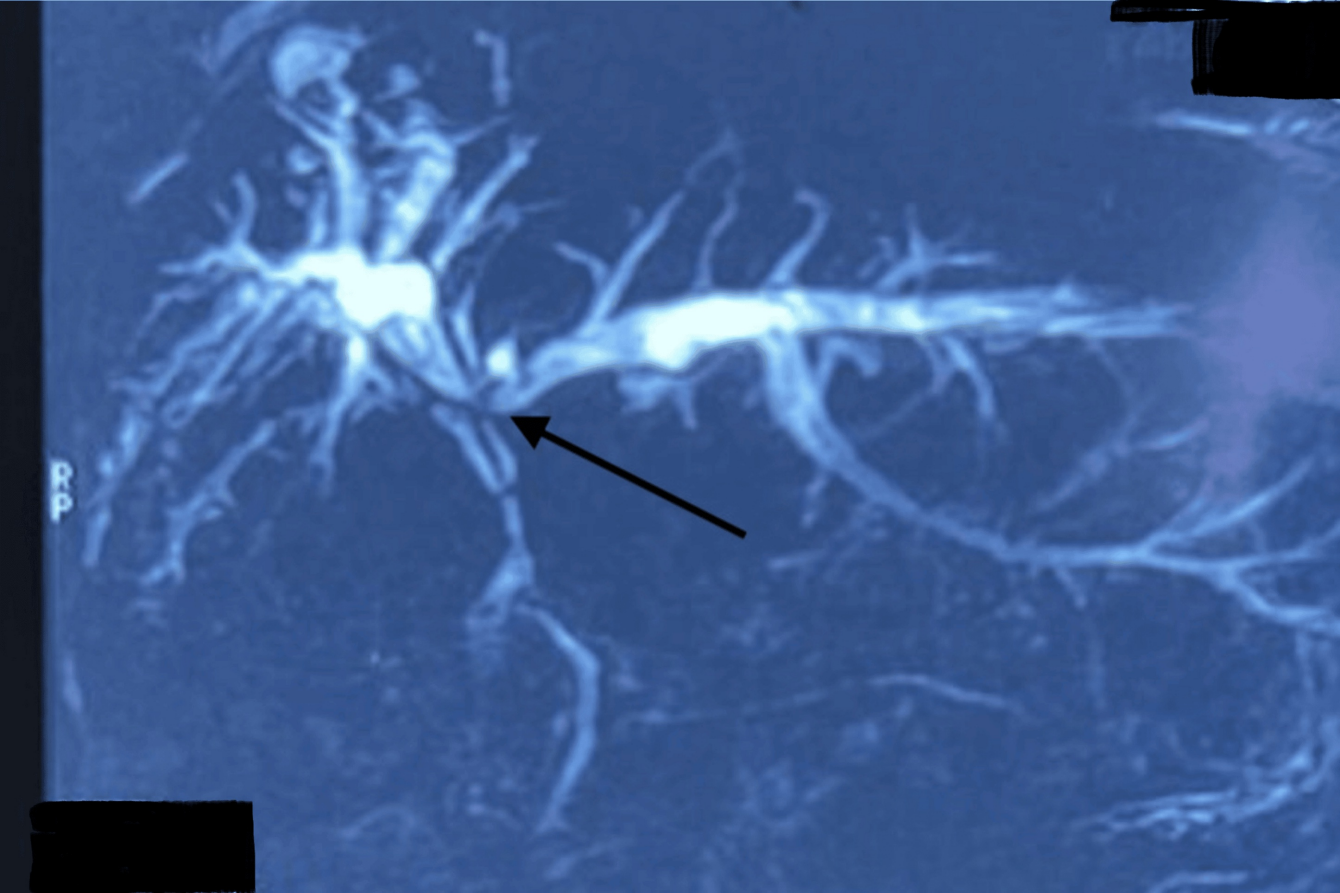
MRCP showing stenosis of hepaticojejunostomy (arrow) and prominence of intrahepatic biliary channels MRCP: magnetic resonance cholangiopancreatography

**Figure 7:**
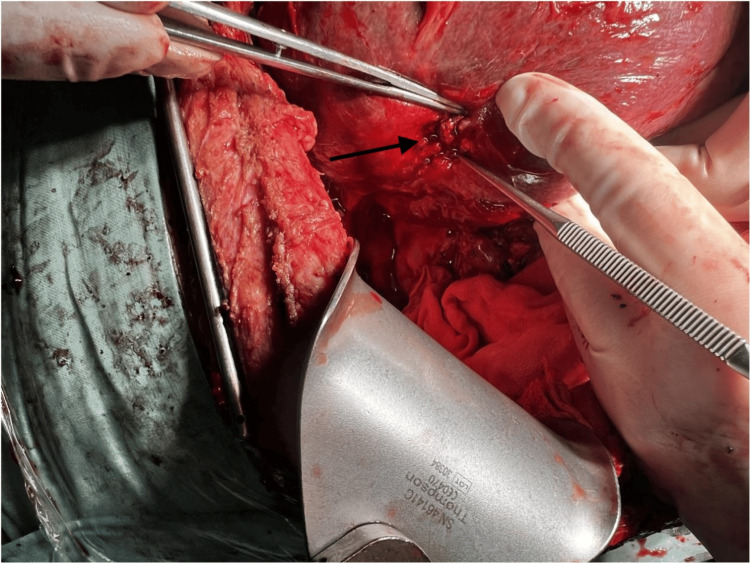
Intraoperative finding showing the fistulous tract arising from segment 7 of the liver (arrow)

Ultrasound and CT imaging, as illustrated in Figures 4, 5, showed an enlarged liver with wavy contours, intrahepatic biliary dilatation, and communication between the right hepatic segments (VII/VIII) and the pleural cavity, suggestive of a biliopleural fistula. Additional findings included right-sided hydropneumothorax, basal consolidation, splenomegaly with venous collaterals, and signs of early cirrhosis. As shown in Figure 6, MRCP further revealed stenosis of hepaticojejunostomy and cholangitic abscesses in the right hepatic lobe, some of which appeared to be communicating with the pleural space. The patient underwent exploratory laparotomy with excision of the biliopleural fistula, revision of the hepaticojejunostomy, and primary repair of the diaphragmatic defect. Figure 7 represents the intraoperative findings, which included a fistulous tract extending from segment VII of the liver through the diaphragm into the pleural cavity, a strictured hepaticojejunostomy, a cholestatic cirrhotic liver, and dense intra-abdominal adhesions. Postoperatively, the patient required intensive care and developed hepatic decompensation attributed to longstanding cholestasis and evolving cirrhosis. She was managed with supportive care, aggressive chest physiotherapy, and incentive spirometry and was transferred out of the ICU on postoperative day 12 and discharged on the 25th postoperative day.

## Discussion

TBF is an uncommon and intricate clinical condition characterized by abnormal connections between the biliary tract and the thoracic cavity or bronchial tree. Peacock first wrote about the condition in 1850 in connection with the rupture of a hydatid cyst. However, it is still very hard to diagnose and treat, even though hepatobiliary surgery and imaging have come a long way [1]. Reported etiologies are diverse, encompassing congenital malformations, trauma, neoplasms, infections such as amoebic or hydatid disease, and, increasingly, iatrogenic causes following hepatobiliary surgery [2,3].

In our series, both patients developed TBF as a consequence of prior biliary surgeries, one subsequent to right hemihepatectomy for a hydatid cyst and the other following stenosis of hepaticojejunostomy, which was done for a common bile duct injury. This underscores the significance of postoperative monitoring in patients with intricate hepatobiliary reconstructions, where delayed fistula formation may arise from chronic inflammation, biliary obstruction, or diaphragmatic injury.

The pathophysiology of TBF is thought to involve subphrenic inflammation or abscess formation, followed by erosion through the diaphragm into the pleural cavity or bronchial tree. When bile enters the thoracic compartment, its irritant effect on pulmonary and pleural tissues sustains inflammation and infection, leading to chronic biliptysis, recurrent pneumonia, and pleural effusion [6,7].

Due to its infrequency, diagnosis is frequently postponed or overlooked. Biliptysis is still the pathognomonic symptom, but lab tests, especially those showing bilirubin in sputum or pleural fluid, can support diagnosis [8-10]. Imaging is crucial: CT defines hepatic and thoracic changes, while MRCP and ERCP enable direct visualization of the fistulous communication and evaluation of biliary obstruction. For defining the anatomy and preparing for surgery in our patients, both MRCP and ERCP were extremely helpful.

Stabilizing the patient with IV fluids, antibiotics, electrolyte correction, nutritional support, chest drainage (if required), and aggressive chest physiotherapy should be the main goals of initial care. After stabilization, definitive therapy ought to be tailored to the patient's overall health and the underlying cause. Decompression through ERCP or percutaneous transhepatic biliary drainage may lower pressure and permit partial fistula closure in cases of biliary obstruction [11]. However, surgical intervention is frequently necessary for persistent or complex fistulae.

The idea behind the surgery is to repair the diaphragmatic and hepatic fistula openings while completely excising the fistulous tract. The location of the fistula, the degree of pulmonary involvement, and the surgeon's experience all influence the surgical technique that is chosen: thoracic, abdominal, or combined thoracoabdominal [12]. Both of the patients in our series had a successful transabdominal fistula excision, which prevented needless pulmonary resection and allowed sufficient exposure for tract division and repair. This strategy is consistent with reports indicating that the abdominal route offers lower morbidity and a shorter recovery period when lung parenchyma is preserved and the disease is benign [13].

Even though our patients have had positive results, TBF still carries a high morbidity and risk of mortality, particularly when combined with cholestasis, infection, or cirrhosis. Management still primarily employs customized, case-based approaches because there are a small number of cases and no randomized data. In order to optimize diagnosis and treatment results, our experience highlights the necessity of interdisciplinary cooperation between thoracic surgeons, interventional radiologists, and hepatobiliary surgeons.

## Conclusions

TBF represent uncommon yet significant complications that require careful consideration, particularly in patients exhibiting biliptysis or bile-stained pleural effusion following hepatobiliary procedures. Timely identification via sputum or pleural bilirubin analysis, coupled with immediate imaging using MRCP or ERCP, can enhance diagnostic precision considerably. Minimally invasive techniques, including endoscopic or percutaneous drainage, may be opted for initially to relieve distal obstruction; however, definitive surgical management is warranted when minimally invasive techniques are unsuccessful.

The findings indicate that an abdominal approach is both safe and effective for benign, non-malignant cases without the need for pulmonary resection. Timely diagnosis, multidisciplinary planning, and individualized operative strategies are fundamental to effective treatment. Multicenter studies are necessary to establish standardized protocols and enhance outcomes for this complex condition.
